# Arthropod Phylogenetics in Light of Three Novel Millipede (Myriapoda: Diplopoda) Mitochondrial Genomes with Comments on the Appropriateness of Mitochondrial Genome Sequence Data for Inferring Deep Level Relationships

**DOI:** 10.1371/journal.pone.0068005

**Published:** 2013-07-15

**Authors:** Michael S. Brewer, Lynn Swafford, Chad L. Spruill, Jason E. Bond

**Affiliations:** 1 Department of Biology, East Carolina University, Greenville, North Carolina, United States of America; 2 Department of Environmental Science, Policy, and Management and Essig Museum of Entomology, University of California, Berkeley, California, United States of America; 3 Department of Biological Sciences and Auburn University Museum of Natural History, Auburn University, Auburn, Alabama, United States of America; Sars International Centre for Marine Molecular Biology, Norway

## Abstract

**Background:**

Arthropods are the most diverse group of eukaryotic organisms, but their phylogenetic relationships are poorly understood. Herein, we describe three mitochondrial genomes representing orders of millipedes for which complete genomes had not been characterized. Newly sequenced genomes are combined with existing data to characterize the protein coding regions of myriapods and to attempt to reconstruct the evolutionary relationships within the Myriapoda and Arthropoda.

**Results:**

The newly sequenced genomes are similar to previously characterized millipede sequences in terms of synteny and length. Unique translocations occurred within the newly sequenced taxa, including one half of the *Appalachioria falcifera* genome, which is inverted with respect to other millipede genomes. Across myriapods, amino acid conservation levels are highly dependent on the gene region. Additionally, individual loci varied in the level of amino acid conservation. Overall, most gene regions showed low levels of conservation at many sites. Attempts to reconstruct the evolutionary relationships suffered from questionable relationships and low support values. Analyses of phylogenetic informativeness show the lack of signal deep in the trees (i.e., genes evolve too quickly). As a result, the myriapod tree resembles previously published results but lacks convincing support, and, within the arthropod tree, well established groups were recovered as polyphyletic.

**Conclusions:**

The novel genome sequences described herein provide useful genomic information concerning millipede groups that had not been investigated. Taken together with existing sequences, the variety of compositions and evolution of myriapod mitochondrial genomes are shown to be more complex than previously thought. Unfortunately, the use of mitochondrial protein-coding regions in deep arthropod phylogenetics appears problematic, a result consistent with previously published studies. Lack of phylogenetic signal renders the resulting tree topologies as suspect. As such, these data are likely inappropriate for investigating such ancient relationships.

## Background

Arthropods comprise more of the Earth's nominal biodiversity than any other comparable eukaryotic group [1], [2] with an estimated 3.7 million species in the tropics alone [3]. Despite their diversity and variety of life strategies, many arthropod groups remain woefully underrepresented in the scientific literature. Consequently, we know little about some of the most diverse and abundant animals on the planet. Even the relationships between many of the most basic groups (i.e., arachnids, myriapods, “crustaceans”, and insects) remain ambiguous. One such understudied taxon is the arthropod class Diplopoda, the millipedes. Millipedes are a highly diverse yet poorly studied group of some 12,000 described species organized into 16 orders. With estimates of global species richness ranging from ∼20,000 [4] to ∼80,000 [2], [5], many species remain to be discovered. Additionally, we know little concerning millipede higher-level relationships, ecology, behavior, physiology, and genomic composition. Due to relatively high degrees of morphological homogeneity and questionable homology of many somatic structures, millipede relationships are poorly resolved with fewer than half of all higher taxa delineated on the basis of phylogenetically defined apomorphic characters.

Only recently have molecular phylogenetic techniques been used to reconstruct millipede phylogenies [6]–[18], however, all but three of these studies focused on relationships at the generic or species level. Regier and Shultz investigated relationships with the Myriapoda using two nuclear protein-coding genes, but their results lacked support. Sierwald and Bond [6] represents the first attempt to reconstruct diplopod ordinal relationships using a total evidence approach by combining the molecular dataset of Regier and Shultz [17], [18] with a morphological matrix from Sierwald *et al*. [19]. Pitz and Sierwald [13] investigated the familial relationships within the order Spirobolida. All of these higher-level studies suffer from limited taxon and locus sampling.

The use of full mitochondrial genome sequences to reconstruct deep relationships has been advocated since 1998 [20]. These data have been employed in various taxa including: salamanders [21], [22], gastropods [23], echinoderms [24], and arthropods [25], including investigations of relationships among the various myriapods classes [26], [27]. However, the utility of mitochondrial genomes in reconstructing deep phylogenies has been the subject of ongoing debate [28]–[30]. The phylogenetic signal present in mitochondrial genomes useful for the study of ancient relationships can be affected by a number of factors (recently summarized in Rota-Stabelli *et al*. [31]). In particular, lineage-specific compositional heterogeneity, has been shown to affect the amino acids used in constructing proteins [32], [33]. This issue is prevalent in ecdysozoans where the genomes tend to have a high A-T to G-C ratio, and, as a result, protein sequences show a bias towards codons that contain adenine and thymine bases [32], [34]. Additionally, compositional heterogeneity can exist between the two strands wherein one strand is more A-T rich than the other [35]. If a region of the genome was inverted, the A-T to G-C bias of the opposite strand could cause a shift in the nucleotide sequence of the region towards the composition of the complementary strand [36]. Heterogeneity in A-T proportion and A-T to G-C bias between strands has been shown to confound phylogenetic inference when using mitochondrial genomes [33], [37], [38].

Another source of error when using mitochondrial genomes to reconstruct deep evolutionary relationships stems from accelerated substitution rates. This leads to long-branch attraction (LBA) [39], [40] resulting in strong outgroup dependent effects [30], [41]. Methods to deal with this issue have been suggested and include the use of site-specific models of molecular evolution, increasing taxon sampling to reinforce weakly supported nodes, and removing problematic sites in the data matrix. The CAT model of molecular evolution accounts for site-specific heterogeneity [42] and has been shown to increase the efficacy of mitochondrial genome-based phylogenomics [31], [43]. Talavera & Vila found that removing problematic portions of the alignment improved the resulting topologies [43].

The full mitochondrial genomes of eight myriapods, including three millipedes, have been sequenced. These include *Scutigera coleoptrata* (Linnaeus, 1758) (Chilopoda: Notostigmophora: Scutigeromorpha: Scutigeridae) [27]; *Lithobius forficatus* (Linnaeus, 1758) (Chilopoda: Pleurostigmophora: Lithobiomorpha: Lithobiidae) [44]; *Bothropolys* sp. (Chilopoda: Pleurostigmophora: Lithobiomorpha: Lithobiidae) (Park, direct GenBank submission); *Symphylella* sp. (Symphyla: Scolopendrellidae) [26]; *Scutigerella causeyae* Michelbacher, 1942 (Symphyla: Scutigerellidae) [45]; *Narceus* “*annularis*” (Diplopoda: Chilognatha: Helminthomorpha: Eugnatha: Julifomia: Spirobolida: Spirobolidae) [46]; *Antrokoreana gracilipes* (Verhoeff, 1938) (Diplopoda: Chilognatha: Helminthomorpha: Eugnatha: Julifomia: Julida: Nemasomatidae) [47]; and *Thyropygus* sp. (Diplopoda: Chilognatha: Helminthomorpha: Eugnatha: Julifomia: Spirostreptida: Harpagophoridae) [46]. The previously sequenced millipede mitochondrial genomes are not a complete representation of the class because sampling is from only three orders comprising the superorder Juliformia, orders Spirobolida, Spirostreptida, and Julida.

We report herein sequences for an additional three entire millipede mitochondrial genomes representing the remaining major groups comprising the Helminthomorpha clade, the worm-like millipedes. We combine these genomes with existing sequence data to investigate, for the first time, the evolution of mitochondrial genomes across the Diplopoda using comparative genomic and phylogenetic methods. We also combine these data with 56 additional Ecdysozoa exemplars, spanning the diversity of this major clade, to ascertain the effects of adding three myriapod taxa in an attempt to strengthen the nodes containing these terminals. All of these analyses taken together provide an evolutionary framework for evaluating the appropriateness of using mitochondrial genome sequences to reconstruct deep evolutionary relationships across the arthropod tree of life and provide further examples of the shortcomings associated with mitochondrial phylogenomics.

## Results

### Genome synteny and features

**Figure 1:**
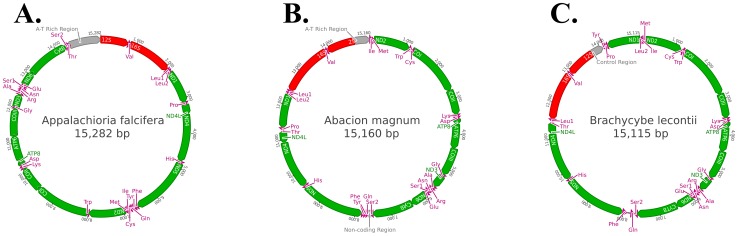
Mitochondrial genomes for the three taxa sequenced as part of this study. A. *Appalachioria falcifera*. B. *Abacion magnum*. C. *Brachycybe leconti*. The grey region corresponds to the A-T Rich Region (the origin of transcription and replication). The red sequences depict the ribosomal subunit DNA. The green regions represent protein-coding sequences. The pink regions correspond to transfer RNAs.

All known myriapod mitochondrial genomes comprise 13 protein-coding regions, two ribosomal subunits, 22 transfer RNAs, at least one non-coding region, and are approximately 15,000 bp in total length. The three genomes sequenced are similar in composition to those previously reported but contain a number of unique features (figure 1). All coding regions are on a single strand in *Appalachioria falcifera* (Keeton, 1959) (Chilognatha: Helminthomorpha: Eugnatha: Merocheta: Polydesmida: Xystodesmidae) (GenBank accession #: JX437063). The regions of *Abacion magnum* Loomis, 1943 (Chilognatha: Helminthomorpha: Eugnatha: Nematophora: Callipodida: Abacionidae) (GenBank accession #: JX437062) are coded half on one strand and half on the other with overlap only in the tRNAs. *Brachycybe lecontii* Wood, 1864 (Chilognatha: Helminthomorpha: Colobognatha: Platydesmida: Andrognathidae) (GenBank accession #: JX437064) is similar to *Ab. magnum* but has a single translocation (ND1) causing a protein-coding region to be on the opposite strand in relation to surrounding regions. All three genomes have undergone tRNA translocations when compared to previously known myriapod sequences. In addition to the previously mentioned translocation of ND1 in *B. lecontii*, *Ap. falcifera* appears to have an entire side of the genome inverted.

All three novel genomes have overlapping gene regions. In *Ap. falcifera*, overlapping occurs between NADH Dehydrogenase protein 4L (*ND4L*) and NADH Dehydrogenase 4 (*ND4*). In *Ab. magnum*, overlapping regions occur between the following gene groups: tRNA-Isoleucine (*Ile*)/tRNA-Methionine (*Met*), tRNA-Tryptophan (*Trp*)/tRNA-Cystine (*Cys*)/Cytochrome Oxidase I (*COI*), ATP synthetase protein 8 (*ATP8*)/ATP synthetase protein 6 (*ATP6*), and tRNA-Asparagine (*Asn*)/tRNA-Serine 1 (*Ser1*). In *B. lecontii*, overlapping occurs involving the following regions: tRNA-Leucine 2 (*Leu2*)/*Ile*/*Met* and *ATP8*/*ATP6*.

All millipede genomes sequenced in the past have included two major non-coding regions. Of the genomes sequenced here, only *Ap. falcifera*, containing a single non-coding region, breaks from this pattern. The genomes are A-T rich, a feature normally seen in arthropods [48]: *Ap. falcifera*  = 64%, *Ab. magnum*  = 66.6%, and *B. lecontii*  = 76.6%.

**Figure 2:**
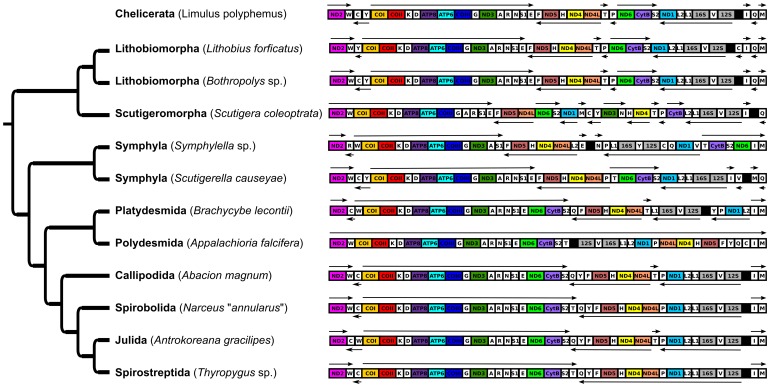
Myriapod mitochondrial genome syntenies depicted in a phylogenetic context. The phylogeny is adapted from Regier and Shultz [18] and Sierwald and Bond [6]. Grey regions are ribosomal subunit genes, white sequences code for transfer RNAs, and black region depicts major A-T Rich region in each genome. The other regions are protein coding; the color scheme is used in subsequent figures.

The mitochondrial genome syntenies are phylogenetically illustrated in figure 2 for all myriapods and the chelicerate *Limulus polyphemus* Müller, 1785, which is considered representative of the ancestral arthropod synteny. The phylogeny on which they are mapped is adapted from the only total evidence analysis of all diplopod orders [6] and mirrors the results obtained herein.

### Protein Coding Region Statistics

**Figure 3:**
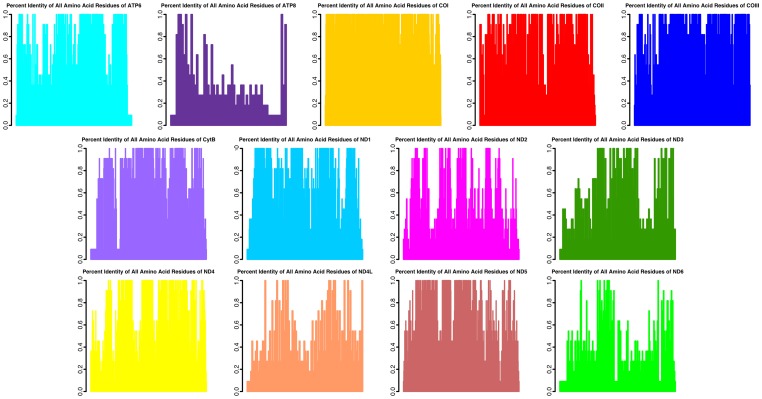
Amino acid conservation values based on identity for each of the 13 protein-coding regions of all currently available myriapod mitochondrial genomes.

**Figure 4:**
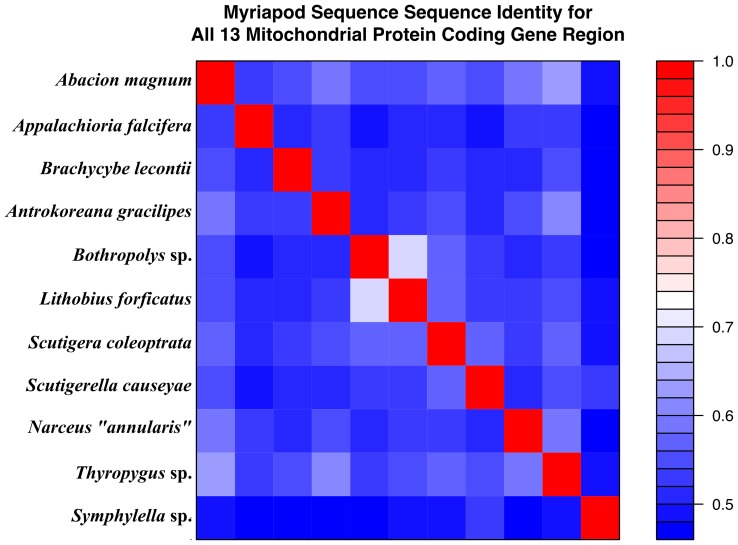
Paiwise comparisons of total mitochondrial protein-coding amino acid percent identity for all myriapod taxasequenced to date. These data show that, overall, taxa do not have high levels of conservation in mitochondrial amino acids sequences. The most similar taxa are the two centipedes of the order Lithobiomorpha and family Lithobiidae, *Lithobius forficatus* and *Bothropolys* sp.

Graphical summaries of the per site amino acid residue conservation score based on identity (AARCI) values for each myriapod gene alignment are shown in figure 3. Pairwise t-tests comparing the AARCIs of each gene region both with and without a Bonferroni correction for multiple tests are summarized in table S2. Of the 78 possible pairwise gene comparisons, 56 are significant in the Bonferroni corrected analysis (71.79%) while 65 are significant in the uncorrected analysis (83.33%). An analysis of variance (ANOVA) indicates differences in the AARCIs between gene regions (F = 67.887, df  = 12, p = 2.2×10^−16^). Pairwise comparisons of percent identity (%ID) based on AARCIs for each myriapod taxon using the concatenated dataset are summarized in figure 4.

### Phylogenetic analyses

**Figure 5:**
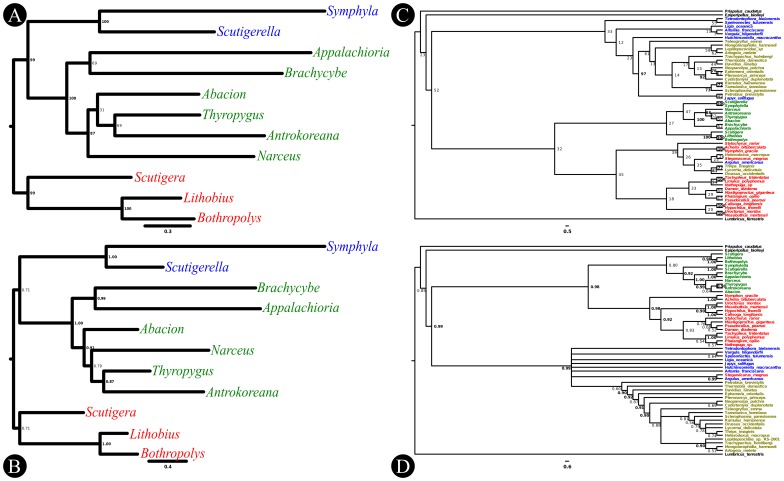
Phylogenetic trees for the Myriapoda and Ecdysozoa based on mitochondrial protein-coding genes. The following phylogenies were reconstructed using maximum likelihood and Bayesian inference of amino acid sequences. The ML trees were obtained using RAxML with 1000 random addition searches followed by 1000 boostrap replicates. The BI trees were obtained from two phylobayes runs consisting of 10000 cycles. The first 2000 cycles were discarded as burn-in. A) Myriapod ML tree, B) myriapod BI tree, C) ecdysozoan ML tree, and D) ecdysozoan BI tree. In the myriapod trees, millipedes taxa are colored green, symphylans are blue, and centipedes are red. In the ecdysozoan trees, outgroup taxa (non-arthropods) are colored black, myriapods are green, chelicerates are red, “crustaceans” and non-insect hexapods are blue, and insects are yellow.

The myriapod phylogenetic analyses are summarized in figure 5A and 5B. The maximum likelihood (ML) and Bayesian Inference (BI) analyses recovered similar topologies differing only in the placement of *Ab. magnum* and *Narceus “annularis”*. The positions of these two taxa are swapped in the two analyses rendering the Juliformia paraphyletic in the ML tree. Overall, the support values are not convincing in either case except for higher-level relationships. The Chilopoda, Pleurostigmophora, Symphyla, and Diplopoda are recovered as monophyletic with strong support in the ML analysis whereas the Pleurostigmophora, Symphyla, Diplopoda, Colobognatha + Polydesmida, and Juliformia + Callipodida are well supported in the BI tree.

The Panarthropoda trees are shown in figure 5C and 5D. The ML tree has a highly improbable topology and very poor support values at most nodes. Monophyletic Ecdysozoa, Panarthropoda, Arthropoda, and Myriapoda are recovered but lack support (BS <70). In general, Pancrustacea taxa are intermingled amongst the chelicerates. The Chilopoda (BS  = 100), Lithobiomorpha (BS  = 100), Diplopoda (BS  = 100), and Symphyla (BS  = 100) are monophyletic with strong support. Within the Chelicerata, the orders Pycnogonida (BS  = 100), Xiphosura (BS  = 100), Scorpiones (BS  = 100), and Araneae (BS  = 100) are monophyletic with strong support along with the Pedipalpi (Amblypygi + Thelyphonida; BS  = 80). Within the Pancrustacea, a clade comprising most of the Hexapoda was recovered with strong support (BS  = 97) but omits a number of taxa that are placed in other groups (e.g., in the chelicerate clade).

The BI panarthropod analysis has poor resolution at some levels but recovered many of the higher groups with moderate support (pp >0.90 unless noted below). The following groups were recovered as monophyletic: Ecdysozoa, Panarthropoda, and Arthropoda, Myriapoda (pp  = 0.80), Chilopoda, Lithobiomorpha, Diplopoda, Pycnogonida, Scorpiones, Araneae, Xiphosura, Insecta, Dicondylia, Pterygota, and Neoptera. The Pancrustacea is paraphyletic and the Chelicerata is polyphyletic as a result of the position of the mite *Steganacarus magnus* (Nicolet, 1855) which groups with the branchiuran *Argulus americanus* Wilson, 1902 (pp  = 0.99). See figure 5D for all supported groupings.

**Figure 6:**
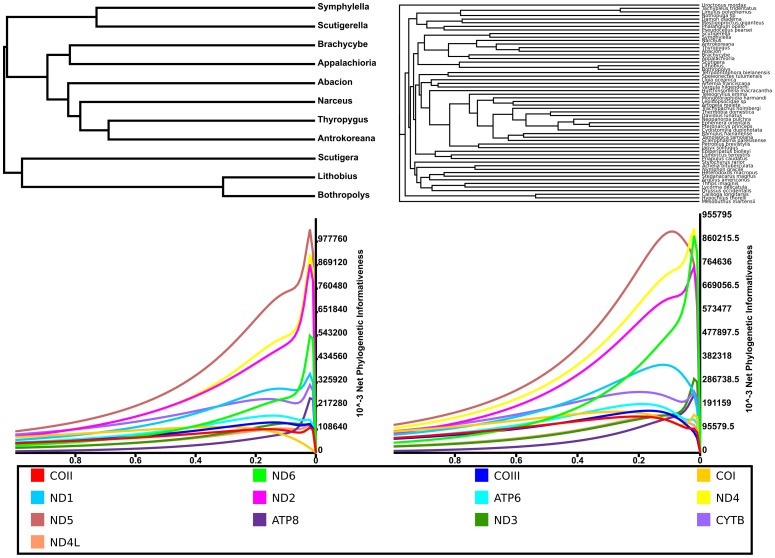
PhyDesign results for all 13 protein-coding mitochondrial gene regions. The peaks for each gene region skewed toward the terminals of both trees. As a result, most signal deep in the trees is confounded by noise. A) Myriapod BI tree converted to ultrametric. B) Ecdysozoan BI tree converted to ultrametric. These results indicate that mitochondrial protein-coding sequences are not appropriate for reconstructing deep arthropod relationships, even when the data is encoded as amino acid residues. The color scheme follows figures 2 and 3.

The PhyDesign analyses, employed to evaluate phylogenetic signal, show a lack of phylogenetic informativeness (PI) for the mitochondrial protein coding genes when used to infer deep arthropod and myriapod relationships. All gene regions have peaked in PI (figure 6) well before the first node back from the tips of the ultrametric trees in both cases.

## Discussion

### Genome synteny and features

The mitochondrial genomes of *Ap. falcifera*, *Ab. magnum*, and *B. lecontii* were similar to the previously sequenced millipede genomes in terms of length, composition, and synteny (figure 1). The synteny of these novel genomes is similar to the juliform millipedes already sequenced with a few exceptions. The translocations of tRNAs is common throughout myriapods and also occurs between the presumably closely related juliform taxa previously sequenced [46], [47]. The genome of *Ab. magnum* is very similar to those of the Juliformia. This is not unexpected; the Callipodida group close to the Juliformia in Sierwald and Bond [6] and in the present study (figure 1). The translocation of *ND1* in *B. lecontii* is the first example of a protein coding gene synteny change in the Diplopoda. The inversion of half of the mitochondrial genome in *Ap. falcifera* is a major rearrangement that is unprecedented in myriapods. Given how anomalous this particular rearrangement is, it was further assessed using novel primers to amplify sequences spanning the inverted section boundaries to confirm this finding.

The synteny of *Ap*. *falcifera* is unique among the currently sequenced millipedes in that all genes appear to be on the same strand (i.e., all genes are transcribed in the same direction). The genomes of *Ab*. *magnum* and *B*. *lecontii*, and all previously analyzed millipedes, have two non-coding regions with most of the genes on either side of the circular genome transcribed in opposite directions and therefore on opposite strands. A mechanism has been previously proposed to explain how two major regions of the genome could have opposite directionality of sense strands [46]. Under this mechanism, the entire genome is duplicated creating a circular genome twice the size and containing two copies of all gene regions and two A-T rich regions (each containing a bi-directional transcription initiator and a bi-directional transcription terminator). Ancestrally, these genes were mixed in terms of which strand was the sense strand because the transcription of each strand could proceed uninterrupted all the way around the circular genome. With the duplication of the A-T rich region, transcription of each strand was terminated at the halfway point. After the duplication, one duplicate of each gene is lost resulting in two halves of the genome transcribed in opposite directions. The directionality of each half of the genome is determined by the position of the translation initiator and terminator sequences (the two remaining non-coding regions found in many millipedes). The process of gene loss is non-random, and, as Lavrov *et al*. [46] point out, could have major implications on the use of mitochondrial genome synteny to reconstruct phylogenies.

Myriapod gene synteny is quite similar to that of *Limulus polyphemus* (figure 2). The lithobiomorph centipedes (*Lithobius* and *Bothropolys*) are identical but for a single tRNA translocation in *Lithobius*. The symphylan *Symphylella* sp. is remarkably different, but *Scutigerella causeyae* is very similar to *Limulus*. All millipedes surveyed have ND6 + CytB placements that differ from that of *Limulus*, and *B*. *lecontii* has a unique positioning of ND1, as mentioned above. These changes are likely associated with the strand specific nature of the opposing halves of millipede mitochondrial genomes. Because each half of diplopod mitochondrial genomes, except for that of *Ap. falcifera,* can only be transcribed in a single direction, those genes on the sense strand of one half of the genome were retained while those on the nonsense strand were lost. Because *Ap*. *falcifera* has a synteny more similar to that of the other millipedes than *Limulus*, taking into account the inversion, and all of the genes are on a single strand, we hypothesize the inversion is a secondary event. *Ap*. *falcifera* likely had a synteny similar to the remaining millipedes and underwent a second inversion event, thus loosing the second non-coding region.

The presence of overlapping regions is not uncommon in taxa studied to date, including the existing myriapod genome sequences [26], [27], [44]–[47]. Recently, White *et al*. [23] found overlapping gene regions in all ten novel pulmonates (Mollusca: Gastropoda). Perseke *et al*. [24] found overlapping genes in the mt-genomes of the ophiurid echinoderms *Ophiocomina nigra* (Abildgaard, 1789) and *Amphipholis squamata* (delle Chiaje, 1828) and in the acorn worm *Balanoglossus clavigerus* (delle Chiaje, 1829) (Hemichordata: Enteropneusta). In the Panarthropoda, overlapping regions exist in the velvet worm *Opisthopatus cinctipes* Purcell, 1899 (Onychophora: Peripatopsidae) [49].

The A-T richness of the genomes reported here are close to the average arthropod level of ∼70% [45]. *Ap. falcifera* (64%) and *Ab. magnum* (66.6%) are lower than *B. lecontii* (76.6%), the highest millipede value to date, but fall within the established range of diplopod A-T contents 62.1% (*Antrokoreana gracilipes*) to 67.8% (*Thyropygus* sp.). Given the paucity of colobognathan genomes sequenced to date and the values of other myriapods (e.g., 72.6%– *Scutigerella causeyae*), it is difficult to say whether *B. lecontii* has abnormally high A-T richness. Other arthropods have even higher A-T content; *Melipona bicolor* (Lepeletier, 1836) (Pancrustacea: Insecta: Hymenoptera) has a value of 86.7% [50].

### Protein Coding Region Statistics

The amino acid residue conservation scores based on identity (AARCIs) suggest inherent differences in the evolution of the protein coding regions of myriapod mitochondrial genomes. The genes show varying levels of site-specific conservation (figure 3). These data indicate some genes with many highly conserved, high AARCI value sites (e.g., *COI*), whereas others have few conserved, low AARCI value sites (e.g., *ATP8*). Additionally, the mean values of AARCI scores differ between the coding regions (table S2). Of the 78 possible pairwise gene comparisons, 56 are significantly different with a Bonferroni correction, whereas 65 are significant in the uncorrected analysis (α = 0.05). An ANOVA comparing the protein coding gene regions show that they are not equal. The taxa also differ in their mean AARCI values across the genome as shown in figure 4, which illustrates the %ID of the amino acid residues for all pairwise comparisons.

Because these taxa have been separated for as long as 504 MY [51], it is not surprising that the gene regions and taxa differ in per site amino acid conservation values. Despite the specific and vital functions performed by these mitochondrial genes, large portions appear to be under varying selection pressures between taxa. Determining whether variable regions are under relaxed or divergent selection will require many more taxa be sequenced to increase phylogenetic coverage. Portions of some genes do appear to be under strong stabilizing selection and probably represent functional domains or important structural regions of the final peptide.

### Phylogenetic analyses

The relationships recovered in the Myriapoda analyses are largely congruent with those of Sierwald and Bond [6], for millipedes, and Regier *et al*. [1], for the myriapod classes (figure 5A and 5B). The relationships recovered in the BI analysis mirror those of Bond and Sierwald [6] and Regier *et al*. [1]. The posterior probabilities and bootstrap values do not indicate strong support at many nodes. The monophyly of the Symphyla and Diplopoda were well supported in both analyses as were the monophyly of the millipede clades Juliformia + Callipodida and Polydesmida + Colobognatha. The ML tree seems to have better support at deeper levels, whereas the BI tree does at shallower nodes. This could be due to the difference in substitution models used in the two analyses or attributed to the vagaries of the optimality criteria employed by each. Taken together, all nodes have strong support from one of the two analyses except the Juliformia and its internal relationships.

Support for a Juliformia + Nematophora (the latter represented here by the Callipodida) grouping agrees with traditional millipede classifications and analyses [6], [52]. The Eugnatha *sensu stricto* was not recovered in our analyses; the Polydesmida allied with the Colobognatha. The presence of a clade comprising the Polydesmida + Colobognatha has been recovered in previous analyses [6], and, if correct, would lend credence to the hypothesis that gonopods are homologous structures across the Colobognatha and Eugnatha clades; a hypothesis that remains up for debate [6]. However, this result could also be due to LBA. More taxa must be sequenced from the Colobognatha and Eugnatha to better test this result.

The ecdysozoan analyses show somewhat different results when comparing the two methods. The ML tree has very low support at most nodes and confuses the relationships of taxa that are confidently placed in monophyletic groups in other studies [1], [53]. Alternatively, the BI analysis recovers established groups more often than the ML analysis. The finding that Bayesian method outperformed likelihood-based approaches is consistent with results reported by Talavera & Vila [43]. However, many nodes remain unresolved and several groups are paraphyletic and/or polyphyletic (e.g., the Arachnida and Pancrustacea as a result of position of *Steganacarus magnus*). Taxon inclusion seems to be very important for breaking up long branches as it appears to lead to better resolution. For example, the Insecta and its nested groupings are well resolved, likely as a consequence of the broad taxon sampling. However, the relationships within are not congruent with existing hypotheses, and the support values borderline strong. The taxa used to reconstruct the ecdysozoan phylogeny were carefully chosen to eliminate unusually divergent taxa that appeared to lead to terminals with considerably longer branches. After working with these data, it has become apparent that taxon selection is crucial when attempting to use mitochondrial protein coding regions to reconstruct deep evolutionary relationships. In a previous study [49] focusing on the Onychophora, the attempts at reconstructing the evolutionary relationships of the Ecdysozoa yielded similarly poor results. Additionally, these ecdysozoan analyses support the existence of the Myriochelata ( = Paradoxopoda; a clade comprising the Chelicerata + Myriapoda). This result contradicts the conventional classification based on morphology where the myriapods are sister to the Pancrustacea ( = Mandibulata). Rota-Stabelli *et al*. [54] were able to recover monophyletic Mandibulata and hypothesized results supporting the Myriochelata were a result of long-branch attraction. Regier *et al*. [1], [55] also recently recovered support for the Mandibulata.

Evidence for why these analyses show low resolution, poorly resolved and conflicting topologies, and low support is evident from the results of the PhyDesign analyses. All genes peak in PI well before the first node back from the tips of the ultrametric trees. The signal behind these peaks is suspect as the effect of noise in the dataset becomes prominent. Based on these data and the fact that some gene regions show little conservation across amino acid sites, it is obvious that mitochondrial genomes are not particularly good markers for deep phylogenetic inference. At the phylogenetic levels investigated herein, there appears to be little phylogenetic signal, and much noise, in the data. Additionally, because mitochondrial genomes experience little to no recombination, a single ancient hybridization event followed by a selective sweep could drastically change the phylogenetic signal contained in mitochondrial DNA data [56]. The use of many unlinked loci from across the nuclear genome would likely be better suited for these types of studies. These nuclear sequences are easily obtainable using transcriptomic sequencing methods such as the Illumina RNAseq technology.

## Conclusions

The genomes of the three millipede taxa sequenced for the first time here are similar in many regards to those previously described. The unique translocation of *ND1* in *B. lecontii* and the inversion of half of the genome in *Ap. falcifera* represent novel and interesting occurrences in the Diplopoda. These results indicate many more unique syntenies may exist across the Diplopoda. Additionally, phylogenetic signal may exist in the genome rearrangements themselves.

Given low levels of amino acid conservation across many regions of the genomes and PhyDesign results, the lack of resolution and confusing topologies produced in our phylogenetic analyses are not surprising. These loci appear to be not particularly well suited for phylogenetic inference at these deep levels (i.e., the relationships between arthropod orders or other higher taxa). Despite the recovery of a myriapod phylogeny similar to those previously published and many reported successes when using mitochondrial genomes to reconstruct deep evolutionary relationships [21]–[24], [26], [45], these data are suspect and should be treated as such. Data from additional sources, such as nuclear protein coding genes, and the use of alignment masking tools along with methods to select genes with good phylogenetic signal, like PhyDesign, should be used in place of mitochondrial protein coding genes when investigating deep arthropod relationships.

## Methods

### Taxon sampling

The three specimens sequenced as part of this analysis were field collected in the southern Appalachian Mountains (table 1). No specific permits were required for the described field studies, the locations were not privately owned or protected, and the study organisms are not endangered or protected. Additional sequences were downloaded from GenBank (table S1). Existing sequences were included for two reasons: 1) to obtain all available myriapod sequences, and 2) additional ecdysozoan sequences were included to represent major lineages (e.g. Priapulida, Onychophora, Chelicerata, and Pancrustacea). An outgroup, Lumbricus terrestris (Linnaeus, 1758) (Annelida: Oligocheata), was chosen from the Lophotrochozoa, the presumed sister-group to the Ecdysozoa.

**Table 1:** Taxonomy and locality data for the specimens sequenced herein.

Class	Order	Family	Species	Country	County	State	Latitude	Longitude
Diplopoda	Platydesmida	Andrognathidae	*Brachycybe lecontii*	USA	Bell	Kentucky	36.72828	−83.72723
Diplopoda	Polydesmida	Xystodesmidae	*Appalachioria falcifera*	USA	Tazewell	Virginia	37.06984	−81.67114
Diplopoda	Callipodida	Abacionidae	*Abacion magnum*	USA	Madison	Alabama	34.74364	−86.51167

### Species identification, vouchers, and molecular methods

Species were identified based on morphological characters and, in the case of *Appalachioria falcifera*, molecular barcodes. *Abacion magnum* was identified by MSB using the characters outlined in [57], CLS identified *Brachycybe lecontii* using the characters of [58], and LS identified *Appalachioria falcifera* as per [9], [11]. Specimen vouchers will be deposited in the Auburn University Museum of Natural History, Auburn, AL and the Field Museum of Natural History, Chicago, Illinois.

Specimens were field collected from the southern Appalachian Mountains (for specific localities, see table 1) and returned to the lab alive. Total DNA was extracted from one individual representing each species using the Qiagen DNEasy Blood and Tissue Kit (Qiagen Inc., Valencia, CA). A portion of the large ribosomal subunit (16S) of the mitochondrial genome was amplified using the universal arthropod primers LR-J-12887 and SR-N-13398 [59]. Unique primers for long amplification of the remainder of the mitochondrial genome were created from within the shorter 16S sequence fragment following Hwang *et al*. [60]: *Ap. falcifera* and *Ab. magnum* (16Saa – 5′ ATG CTA CCT TTG TAC AGT CAA TAT ACT GCA GC 3′; 16Sbb – 5′ CAT ATT GAC AAT AAT GTT TGC GAC CTC GAT GTT 3′) and *B. lecontii* (16Saa – 5′ ATG CTA CCT TCG TAC AGT TAA TAT ACT GCA AC 3′; 16Sbb – 5′ CAT ATT GAT AAA TAA GTT TGT GAC CTC GAT GTT 3′). Takara LAtaq was used with the custom primers to amplify the remainder of the mitochondrial genome following the manufacturers recommended protocols. The resulting amplicons were approximately 15,000 bp in length. The genome of *Ap*. *falcifera* was sequenced following the methods of Swafford and Bond [61]. The genomes of *Ab. magnum* and *B. lecontii* were sequenced as follows. The long PCR products were fragmented using the Roche GS FLX Standard Nebulizers Kit. Fragments approximately 500 bp in length were selected via electrophoresis in an Agarose gel and extracted. The extracted DNA fragments were end repaired and cloned using the Zero Blunt PCR Cloning Kit (Invitrogen, Carlsbad, CA). Individual colonies were selected, and the plasmid inserts were amplified following the manufacturers recommendations. PCR products were purified using ExoSAP-IT (USB, Cleveland, OH) and sequenced with an ABI Prism 3730 automated DNA sequencer (Applied Bio-systems, Foster City, CA) using ABI Big Dye Terminator version 3.2 Cycle Sequencing Ready Reaction Kit purified with Sephadex G-50 (Sigma-Aldrich, St. Louis, MO).

### Genome assembly and annotation

The resulting sequence reads were scanned for plasmid contamination, quality trimmed, manually edited, and assembled into contigs using Geneious version 5.5 (Biomatters Ltd, Auckland, New Zealand). Novel primers were designed to bridge any gaps between contigs using BLAST annotations and the existing millipede mitochondrial genome syntenies as a guide. Final annotations were performed in Geneious by identifying open reading frames (ORFs) and confirming the annotations with BLAST searches. The ORFs were adjusted to account for the surrounding gene boundaries using alternative starts and the completion of TAA stop codons following polyadenylation. Transfer RNA sequences (tRNA) were identified using tRNAscan-SE 1.21 [62] followed by a modified version [63] to account for difficult to find regions. The ribosomal subunits (12 S and 16 S) were identified using the existing myriapod sequences, and the non-coding regions were delineated as the remaining unannotated sequences. The translated ORFs were used in subsequent analyses.

### Statistics and phylogenetic analyses

Amino acid (AA) sequences are used instead of nucleotides because AA changes tend to evolve more slowly due to the redundancy of the genetic code thus resisting per site saturation of changes over time. Sequences representing each protein coding region for all sequenced myriapods (Table S1) were aligned using MAFFT version 6 [64], [65]. The amino acid residue conservation values of these alignments were calculated using the Bio3D package in R [66]. The amino acid residue conservation value based on identity scores (AARCIs) for each position in an alignment were calculated as the average identity score for all possible pairwise comparisons using the command “conserv” with “method  =  ‘identity’”. These AARCI values were also calculated for a concatenated dataset consisting of all translated protein coding gene region alignments. Using the AARCIs, pairwise comparisons between each taxon for each gene region alignment and the concatenated dataset were conducted. Pairwise t-tests were conducted comparing amino acid residue conservation values of each gene to all others with a Bonferroni p-value adjustment and without (table S2) using the R command pairwise.t.test as implemented in the “stats” package [67]. Any gaps are not missing data but should represent actual insertions and/or deletions. An analysis of variance (ANOVA) comparing the individual gene regions was conducted using the “aov” command in R.

A supermatrix consisting of all amino acid residue alignments for the myriapods and for the Ecdysozoa was used to infer evolutionary relationships. Analyses were performed under maximum likelihood (ML) and Bayesian inference (BI) optimality criteria using the computer programs RAxML 7.2.8 [68] and Phylobayes 3.3 b [69] respectively. The ML analyses consisted of 1000 random addition sequence (RAS) searches followed by 1000 bootstrap (BS) replicates that were applied to the best tree from the RAS analyses. The myriapod ML analysis was conducted under the arthropod mitochondrial (MTART) model of molecular evolution while the ecdysozoan analysis employed the CAT model. The BI analyses consisted of two independent run of 10,000 cycles, sampled every cycle, and used the default model (CAT) and parameters. The BI consensus tree was obtained from both runs with the first 2,000 cycles discarded as burn-in. The maxdiff value for the myriapod analysis was reported below 0.1 (0.0445), indicating a good run. The maxdiff value for the panarthropod analysis was reported below 0.3 (0.24425), indicating an acceptable run.

Analyses were attempted using the CAT-BP model as implemented in NH-PhyloBayes [70]. This model was developed to account for site- and lineage-specific heterogeneity in amino acid substitutions. Unfortunately after millions of generations, convergence between runs was not reached with either the millipede or ecdysozoan dataset. Individual runs resulted trees with topologies similar to those shown herein but lacked high support values.

Phylogenetic informativeness was calculated for the individual protein coding gene regions using the online program PhyDesign [71]. For these analyses, the fully resolved ML trees were converted into ultrametric trees using the command “chronopl” as implemented in the R package APE [72]. The results are summarized in figure 6.

## Supporting Information

Table S1 Specimens and sequences examined as part of our investigations.
(DOCX)

Table S2 Comparison of average myriapod amino acid conservation values for all 13 protein-coding amino acid alignements.
(DOCX)
