# Supplementary material for: Arthropod Phylogenetics in Light of Three Novel Millipede (Myriapoda: Diplopoda) Mitochondrial Genomes with Comments on the Appropriateness of Mitochondrial Genome Sequence Data for Inferring Deep Level Relationships
Source: PLoS One. 2013 Jul 15;8(7):e68005. doi: 10.1371/journal.pone.0068005 (PMC3712015; doi:10.1371/journal.pone.0068005)
Supplement: Table S1 — Specimens and sequences examined as part of our investigations. (DOCX) [file pone.0068005.s001.docx]

## Table S1 - Specimens and sequences examined as part of our investigations.

| **Phylum** | **Class** | **Order** | **Species** | **Accession Number** | **Authors** | **Journal** | **Year** |
| --- | --- | --- | --- | --- | --- | --- | --- |
| Annelida | Clitellata | Haplotaxida | *Lumbricus terrestris* | NC_001673 | Boore,J.L. and Brown,W.M. | Genetics 141 (1), 305-319 | 1995 |
| Priapulida |  |  | *Priapulus caudatus* | NC_008557 | Webster,B.L. *et al*. | Evol. Dev. 8 (6), 502-510 | 2006 |
| Onychopora |  |  | *Epiperipatus biolleyi* | NC_009082 | Podsiadlowski,L., Kohlhagen,H. and Koch,M. | Mol. Phylogenet. Evol. 45 (1), 251-260 | 2007 |
| Arthropoda | Chilopoda | Scutigeromorpha | *Scutigera coleoptrata* | AJ_507061 | Negrisolo, A., Minelli, A., and Valle, G. | Molecular Biology and Evolution 21 (4), 770-780 | 2004 |
| Arthropoda | Chilopoda | Lithobiomorpha | *Lithobius* | AJ_507061 | Negrisolo, A., Minelli, A., and Valle, G. | Molecular Biology and Evolution 21 (4), 770-780 | 2004 |
| Arthropoda | Chilopoda | Lithobiomorpha | *Bothropolys* | NC_005870 | Lavrov,D.V., Brown,W.M. and Boore,J.L. | Proc. Natl. Acad. Sci. U.S.A. 97 (25), 13738-13742 | 2000 |
| Arthropoda | Symphyla |  | *Symphylella* | NC_009458 | Park, S. | Unpublished |  |
| Arthropoda | Symphyla |  | *Scutigerella* | NC_011572 | Gai,Y. *et al*. | Mol. Phylogenet. Evol. 49 (2), 574-585 | 2008 |
| Arthropoda | Diplopoda | Platydesmida | *Brachycybe* | JX437064 | Podsiadlowski,L., Kohlhagen,H. and Koch,M. | Mol. Phylogenet. Evol. 49 (2), 574-585 | 2007 |
| Arthropoda | Diplopoda | Polydesmida | *Appalachioria* | JX437063 | Brewer, M.S., Swafford, L., Spruill, C., and Bond, J.E. |  |  |
| Arthropoda | Diplopoda | Spirobolida | *Narceus* | NC_003343 | Lavrov,D.V., Boore,J.L. and Brown,W.M. | Mol. Biol. Evol. 19 (2), 163-169 | 2002 |
| Arthropoda | Diplopoda | Spirostreptida | *Thyropygus* | NC_003343 | Lavrov,D.V., Boore,J.L. and Brown,W.M. | Mol. Biol. Evol. 19 (2), 163-169 | 2002 |
| Arthropoda | Diplopoda | Julida | *Antrokoreana* | NC_010221 | Woo,H.J. *et al*. | Mol. Cells 23 (2), 182-191 | 2007 |
| Arthropoda | Diplopoda | Callipodida | *Abacion* | JX437062 | Brewer, M.S., Swafford, L., Spruill, C., and Bond, J.E. |  |  |
| Arthropoda | Pycnogonida | Pantopoda | *Nymphon gracile* | NC_008572 | Podsiadlowski,L. and Braband,A. | BMC Genomics 7, 284 | 2006 |
| Arthropoda | Pycnogonida | Pantopoda | *Achelia bituberculata* | NC_009724 | Park,S.J., Lee,Y.S. and Hwang,U.W. | BMC Genomics 8, 343 | 2007 |
| Arthropoda | Arachnida | Scorpiones | *Uroctonus mordax* | NC_010782 | Masta,S.E. and Boore,J.L. | Mol. Biol. Evol. 25 (5), 949-959 | 2008 |
| Arthropoda | Arachnida | Scorpiones | *Mesobuthus martensii* | NC_009738 | Choi,E.H. *et al*. | DNA Seq. 18 (6), 461-473 | 2007 |
| Arthropoda | Arachnida | Araneae | *Hypochilus thorelli* | NC_010777 | Masta,S.E. and Boore,J.L. | Mol. Biol. Evol. 25 (5), 949-959 | 2008 |
| Arthropoda | Arachnida | Araneae | *Calisoga longitarsis* | NC_010780 | Masta,S.E. and Boore,J.L. | Mol. Biol. Evol. 25 (5), 949-959 | 2008 |
| Arthropoda | Arachnida | Mesostigmata | *Stylochyrus rarior* | NC_013474 | Swafford,L. and Bond,J.E. | Invertebr. Syst. 23 (5), 445-451 | 2009 |
| Arthropoda | Arachnida | Sarcoptiformes | *Steganacarus magnus* | NC_011574 | Simmons,D.G. *et al*. | BMC Genomics 9, 352 | 2008 |
| Arthropoda | Arachnida | Thelyphonida | *Mastigoproctus giganteus* | NC_010430 | Masta,S.E. and Boore,J.L. | Mol. Biol. Evol. 25 (5), 949-959 | 2008 |
| Arthropoda | Arachnida | Ricinulei | *Pseudocellus pearsei* | NC_009985 | Fahrein,K. *et al*. | BMC Genomics 8, 386 | 2007 |
| Arthropoda | Arachnida | Amblypygi | *Damon diadema* | NC_011293 | Fahrein,K., Masta,S.E. and Podsiadlowski,L. | Genome 52 (5), 456-466 | 2009 |
| Arthropoda | Merostomata | Xiphosura | *Tachypleus tridentatus* | NC_012574 | Weng,Z.-H. *et al*. | Unpublished |  |
| Arthropoda | Merostomata | Xiphosura | *Limulus polyphemus* | NC_003057 | Lavrov,D.V., Boore,J.L. and Brown,W.M. | Mol. Biol. Evol. 17 (5), 813-824 | 2000 |
| Arthropoda | Arachnida | Opiliones | *Phalangium opilio* | NC_010766 | Masta,S.E. and Boore,J.L. | Mol. Biol. Evol. 25 (5), 949-959 | 2008 |
| Arthropoda | Arachnida | Solifugae | *Nothopuga sp.* | NC_009984 | Fahrein,K. *et al*. | BMC Genomics 8, 386 | 2007 |
| Arthropoda | Ellipura | Collembola | *Tetrodontophora bielanensis* | NC_002735 | Nardi,F. *et al*. | Mol. Biol. Evol. 18 (7), 1293-1304 | 2001 |
| Arthropoda | Ostracoda | Mycodocopida | *Vargula hilgendorfii* | NC_005306 | Ogoh,K. and Ohmiya,Y. | Gene 327 (1), 131-139 | 2004 |
| Arthropoda | Remipedia | Nectiopoda | *Speleonectes tulumensis* | NC_005938 | Lavrov,D.V., Brown,W.M. and Boore,J.L. | Proc. R. Soc. Lond., B, Biol. Sci. 271 (1538), 537-544 | 2004 |
| Arthropoda | Malacostraca | Isopoda | *Ligia oceanica* | NC_008412 | Kilpert,F. and Podsiadlowski,L. | BMC Genomics 7, 241 | 2006 |
| Arthropoda | Diplura |  | *Japyx solifugus* | NC_007214 | Carapelli,A. *et al*. | Crustac. Issues 16, 295-306 | 2005 |
| Arthropoda | Cephalocarida | Brachypoda | *Hutchinsoniella macracantha* | NC_005937 | Lavrov,D.V., Brown,W.M. and Boore,J.L. | Proc. R. Soc. Lond., B, Biol. Sci. 271 (1538), 537-544 | 2004 |
| Arthropoda | Branchippoda | Anostraca | *Artemia franciscana* | NC_001620 | Perez,M.L. *et al*. | J. Mol. Evol. 38 (2), 156-168 | 1994 |
| Arthropoda | Maxillopoda | Arguloida | *Argulus americanus* | NC_005935 | Lavrov,D.V., Brown,W.M. and Boore,J.L. | Proc. R. Soc. Lond., B, Biol. Sci. 271 (1538), 537-544 | 2004 |
| Arthropoda | Insecta | Archaeognatha | *Petrobius brevistylis* | NC_007688 | Podsiadlowski,L. | Insect Mol. Biol. 15 (3), 253-258 | 2006 |
| Arthropoda | Insecta | Thysanura | *Thermobia domestica* | NC_006080 | Cook,C.E., Yue,Q. and Akam,M. | Proc. Biol. Sci. 272 (1569), 1295-1304 | 2005 |
| Arthropoda | Insecta | Odonata | *Davidius lunatus* | NC_012644 | Kim,I. *et al*. | Unpublished |  |
| Arthropoda | Insecta | Ephemeroptera | *Ephemera orientalis* | NC_012645 | Kim,I. *et al*. | Unpublished |  |
| Arthropoda | Insecta | Plecoptera | *Pteronarcys princeps* | NC_006133 | Stewart,J.B. and Beckenbach,A.T. | Genome 49 (7), 815-824 | 2006 |
| Arthropoda | Insecta | Mecoptera | *Neopanorpa pulchra* | NC_013180 | Hua,J. *et al*. | Unpublished |  |
| Arthropoda | Insecta | Diptera | *Cydistomyia duplonotata* | NC_008756 | Cameron,S.L. *et al*. | Syst. Entomol. 32 (1), 40-59 | 2007 |
| Arthropoda | Insecta | Orthoptera | *Teleogryllus emma* | NC_011823 | Ye,W., Pan,C. and Huang,Y. | Unpublished |  |
| Arthropoda | Insecta | Mantodea | *Tamolanica tamolana* | NC_007702 | Cameron,S.L., Barker,S.C. and Whiting,M.F. | Mol. Phylogenet. Evol. 38 (1), 274-279 | 2006 |
| Arthropoda | Insecta | Mantophasmatodea | *Sclerophasma paresisense* | NC_007701 | Cameron,S.L., Barker,S.C. and Whiting,M.F. | Mol. Phylogenet. Evol. 38 (1), 274-279 | 2006 |
| Arthropoda | Insecta | Phasmatodea | *Ramulus hainanense* | NC_013185 | Hua,J. *et al*. | Unpublished |  |
| Arthropoda | Insecta | Hymenoptera | *Orussus occidentalis* | NC_012689 | Dowton,M. *et al*. | Mol. Biol. Evol. 26 (7), 1607-1617 | 2009 |
| Arthropoda | Insecta | Hemiptera | *Lycorma delicatula* | NC_012835 | Hua,J. *et al*. | BMC Evol. Biol. 9, 134 | 2009 |
| Arthropoda | Insecta | Thysanoptera | *Thrips imaginis* | NC_004371 | Shao,R. and Barker,S.C. | Mol. Biol. Evol. 20 (3), 362-370 | 2003 |
| Arthropoda | Insecta | Phthiraptera | *Heterodoxus macropus* | NC_002651 | Shao,R., Campbell,N.J. and Barker,S.C. | Mol. Biol. Evol. 18 (5), 858-865 | 2001 |
| Arthropoda | Insecta | Psocoptera | Lepidopsocidae sp. *RS-2001* | NC_004816 | Shao,R. and Barker,S.C. | Mol. Biol. Evol. 20 (3), 362-370 | 2003 |
| Arthropoda | Insecta | Coleoptera | *Trachypachus holmbergi* | NC_011329 | Sheffield,N.C. *et al*. | Mol. Biol. Evol. 25 (11), 2499-2509 | 2008 |
| Arthropoda | Insecta | Raphidioptera | *Mongoloraphidia harmandi* | NC_013251 | Cameron,S.L. *et al*. | Zool. Scr. | 2009 |
| Arthropoda | Insecta | Lepidoptera | *Artogeia melete* | NC_010568 | Yang,L. *et al*. | Mol. Biol. Rep. 36 (6), 1441-1449 | 2009 |
