# Supplementary material for: Arthropod Phylogenetics in Light of Three Novel Millipede (Myriapoda: Diplopoda) Mitochondrial Genomes with Comments on the Appropriateness of Mitochondrial Genome Sequence Data for Inferring Deep Level Relationships
Source: PLoS One. 2013 Jul 15;8(7):e68005. doi: 10.1371/journal.pone.0068005 (PMC3712015; doi:10.1371/journal.pone.0068005)
Supplement: Table S2 — Comparison of average myriapod amino acid conservation values for all 13 protein-coding amino acid alignements. (DOCX) [file pone.0068005.s002.docx]

## Table S2 - Comparison of average myriapod amino acid conservation values for all 13 protein-coding amino acid alignements.

| **Uncorrected p-values** | | |  |  |  |  |  |  |  |  |  |  |
| --- | --- | --- | --- | --- | --- | --- | --- | --- | --- | --- | --- | --- |
|  | **ATP6** | **ATP8** | **COI** | **COII** | **COIII** | **CytB** | **ND1** | **ND2** | **ND3** | **ND4** | **ND4L** | **ND5** |
| **ATP8** | **6.84E-11** | -- | -- | -- | -- | -- | -- | -- | -- | -- | -- | -- |
| **COI** | **3.69E-21** | **2.05E-35** | -- | -- | -- | -- | -- | -- | -- | -- | -- | -- |
| **COII** | **0.0004** | **1.26E-18** | **1.58E-07** | -- | -- | -- | -- | -- | -- | -- | -- | -- |
| **COIII** | **5.70E-05** | **6.30E-20** | **4.07E-07** | 0.7322 | -- | -- | -- | -- | -- | -- | -- | -- |
| **CytB** | 0.6690 | **4.44E-11** | **6.45E-32** | **8.49E-06** | **5.11E-07** | -- | -- | -- | -- | -- | -- | -- |
| **ND1** | 0.5988 | **1.68E-10** | **2.41E-28** | **1.36E-05** | **1.08E-06** | 0.8895 | -- | -- | -- | -- | -- | -- |
| **ND2** | **4.54E-17** | 0.0936 | **8.89E-96** | **2.31E-34** | **5.39E-39** | **1.17E-20** | **8.95E-18** | -- | -- | -- | -- | -- |
| **ND3** | 0.0395 | **7.72E-06** | **9.19E-22** | **6.26E-07** | **8.08E-08** | 0.0583 | 0.0825 | **7.90E-06** | -- | -- | -- | -- |
| **ND4** | **0.0066** | **1.26E-07** | **7.90E-50** | **1.16E-11** | **8.41E-14** | **0.0069** | **0.0179** | **3.64E-12** | 0.8922 | -- | -- | -- |
| **ND4L** | **0.0002** | **0.0032** | **3.95E-27** | **9.84E-11** | **7.68E-12** | **0.0002** | **0.0004** | **0.0285** | 0.1056 | **0.0351** | -- | -- |
| **ND5** | **0.0001** | **1.90E-06** | **2.27E-65** | **6.59E-16** | **8.23E-19** | **3.94E-05** | **0.0003** | **7.91E-10** | 0.5130 | 0.1985 | 0.1544 | -- |
| **ND6** | **6.11E-20** | 0.8196 | **7.60E-80** | **2.45E-35** | **5.68E-39** | **1.32E-22** | **2.35E-20** | **0.0280** | **1.30E-08** | **4.08E-15** | **0.0004** | **6.51E-13** |
|  |  |  |  |  |  |  |  |  |  |  |  |  |
| **Bonferroni corrected p-values** | | | |  |  |  |  |  |  |  |  |  |
|  | **ATP6** | **ATP8** | **COI** | **COII** | **COIII** | **CytB** | **ND1** | **ND2** | **ND3** | **ND4** | **ND4L** | **ND5** |
| **ATP8** | **5.33E-09** | -- | -- | -- | -- | -- | -- | -- | -- | -- | -- | -- |
| **COI** | **2.88E-19** | **1.60E-33** | -- | -- | -- | -- | -- | -- | -- | -- | -- | -- |
| **COII** | **0.0279** | **9.79E-17** | **1.23E-05** | -- | -- | -- | -- | -- | -- | -- | -- | -- |
| **COIII** | **0.0044** | **4.91E-18** | **3.18E-05** | 1.0000 | -- | -- | -- | -- | -- | -- | -- | -- |
| **CytB** | 1.0000 | **3.46E-09** | **5.03E-30** | **0.0007** | **3.98E-05** | -- | -- | -- | -- | -- | -- | -- |
| **ND1** | 1.0000 | **1.31E-08** | **1.88E-26** | **0.0011** | **8.39E-05** | 1.0000 | -- | -- | -- | -- | -- | -- |
| **ND2** | **3.54E-15** | 1.0000 | **6.93E-94** | **1.81E-32** | **4.21E-37** | **9.10E-19** | **6.98E-16** | -- | -- | -- | -- | -- |
| **ND3** | 1.0000 | **0.0006** | **7.16E-20** | **4.88E-05** | **6.30E-06** | 1.0000 | 1.0000 | **0.0006** | -- | -- | -- | -- |
| **ND4** | 0.5177 | **9.85E-06** | **6.16E-48** | **9.08E-10** | **6.56E-12** | 0.5365 | 1.0000 | **2.84E-10** | 1.0000 | -- | -- | -- |
| **ND4L** | **0.0137** | 0.2490 | **3.08E-25** | **7.67E-09** | **5.99E-10** | **0.0163** | **0.0341** | 1.0000 | 1.0000 | 1.0000 | -- | -- |
| **ND5** | **0.0089** | **0.0001** | **1.77E-63** | **5.14E-14** | **6.42E-17** | **0.0031** | **0.0215** | **6.17E-08** | 1.0000 | 1.0000 | 1.0000 | -- |
| **ND6** | **4.77E-18** | 1.0000 | **5.93E-78** | **1.91E-33** | **4.43E-37** | **1.03E-20** | **1.84E-18** | 1.0000 | **1.02E-06** | **3.18E-13** | **0.0284** | **5.08E-11** |

The top half of the table show the results of uncorrected pairwise t-tests between each gene. The lower half shows Bonferroni corrected results. Bolded values are significant (alpha = 0.05).
